# Clinicopathological pitfalls associated with benign uterine mesenchymal tumors: A single-center experience

**DOI:** 10.1097/MD.0000000000042852

**Published:** 2025-06-13

**Authors:** Shizuka Iwabuchi, Yusuke Sato, Mamiko Okamoto, Mitsutake Yano, Eiji Kobayashi

**Affiliations:** a Department of Obstetrics and Gynecology, Faculty of Medicine, Oita University, Oita, Japan; b Department of Obstetrics and Gynecology, Oita Prefectural Hospital, Oita, Japan.

**Keywords:** cotyledonoid-dissecting leiomyoma, metastasizing leiomyoma, torsion, uterine mesenchymal tumor, uterine tumor resembling ovarian sex-cord tumor

## Abstract

Diagnosis of uterine mesenchymal tumors continues to be challenging because of their nonspecific clinicopathological presentation. Several studies have focused on the underdiagnosis/undertreatment of hidden uterine sarcomas. However, few have examined the overdiagnosis/overtreatment of benign uterine mesenchymal tumors that masquerade as uterine sarcomas. We report 4 cases of benign uterine mesenchymal tumors that were preoperatively diagnosed as having malignant potential and underwent extensive surgery. The patients had cotyledonoid-dissecting leiomyomas, uterine tumors resembling ovarian sex-cord tumors, metastasizing leiomyomas, and torsion of a subserosal uterine leiomyoma. The patients’ ages ranged from 42 to 59 years (median 51.5). All 4 cases were suspected of having a malignant tumor based on preoperative clinical imaging, and 1 case was suspected of having a malignant tumor based on preoperative imaging and pathological evaluations of biopsy. All patients underwent surgery, including a hysterectomy. One of the 4 patients (25%) underwent lymphadenectomy, and 1 (25%) underwent partial lung resection. All patients survived without evidence of disease. Overall, detailed pre- and intraoperative clinical and pathological evaluations may be insufficient for diagnosis. Physicians should be aware of the diversity of uterine mesenchymal tumors, the difficulty in diagnosing them, and how to avoid these pitfalls.

## 1. Introduction

Uterine mesenchymal tumors continue to be challenging to diagnose owing to their nonspecific clinical presentation, often non-distinctive gross appearance, varied (and overlapping) morphological appearance, and unsuspected pitfalls in immunohistochemical expression.^[1]^ Even benign mesenchymal tumors of the uterus can be life-threatening owing to the accompanying symptoms^[2]^ and can undergo varied metastases over time.^[3]^ The US Food and Drug Administration estimates that a hidden uterine mesenchymal malignancy/sarcoma may be present in approximately 1 in 225 to 1 in 580 women undergoing surgery for uterine fibroids.^[4]^ Several studies have focused on the underdiagnosis/undertreatment of hidden uterine sarcomas^[5,6]^; however, few have focused on the overdiagnosis/overtreatment of benign uterine mesenchymal tumors that masquerade as uterine sarcomas.^[7]^ Herein, we report 4 cases of benign uterine mesenchymal tumors that were preoperatively diagnosed as having malignant potential and underwent extensive surgery.

## 2. Case presentations

The cases in the present study were identified based on diagnostic uncertainty among patients who underwent surgery for gynecologic diseases at Oita University Hospital between 2020 and 2023. Specifically, these were cases in which a malignant tumor was diagnosed preoperatively, but a benign tumor was diagnosed postoperatively. Written informed consent for publication was obtained. These 4 cases are summarized in Table 1. The patients’ ages ranged from 42 to 59 years (median 51.5). All 4 cases were suspected of having a malignant tumor based on preoperative clinical imaging, and 1 case was suspected of having a malignant tumor based on preoperative imaging and pathological evaluations of biopsy. All patients underwent surgery, including a hysterectomy. One of the 4 patients (25%) underwent lymphadenectomy, and 1 (25%) underwent partial lung resection. The operative time was 158 to 371 minutes (median 164 minutes), and intraoperative bleeding was 10 to 1600 mL (median 410 mL). The postoperative pathological diagnosis revealed that none of the patients had malignant tumors. All patients survived without evidence of disease. The details of these cases are presented below.

**Table 1:** Summary of the present 4 cases.

	Case 1	Case 2	Case 3	Case 4
Age (yr)	42	59	53	50
Gravidity	0	2	4	3
Parity	0	2	3	2
Menopause (age) (yr)	42	44	42	49
Chief complaint	Lower abdominal pain	Abnormal genital bleeding	None	Abdominal fullness
Cervical cytology	Negative	Atypical glandular cells	Post-hysterectomy	Negative
Endometrial cytology/biopsy	Negative	Suspicious of carcinosarcoma	Post-hysterectomy	Negative
Preoperative diagnosis	Uterine sarcoma	Endometrial carcinosarcoma	Lung cancer	Ovarian cancer
Postoperative diagnosis	Cotyledonoid-dissecting leiomyoma	UTROSCT	Benign metastatic uterine leiomyoma	Uterine leiomyoma
Surgery	TAH + BSO	TAH + BSO + retoroperitoneal lymphadenectomy + omentecotmy	Partial right lung resection	TAH + BSO
Surgical time (min)	170	371	115	158
Surgical blood loss (mL)	240	1600	10	580
Comorbidity	Mental retardation, epilepsy	Hypertension	None	None
Clinical history	None	Subarachnoid hemorrhage	TAH for uterine leiomyoma	Colorectal cancer, myomectomy of uterine leiomyoma
Family history	Diabetes, gastric cancer	Gastric cancer, subarachnoid hemorrhage	Lung cancer	None
Outcome	DFS 1 month after surgery	DFS 17 months after surgery	DFS 12 months after surgery	DFS 1 month after surgery

BSO = bilateral salpingo-oophorectomy, DFS = disease-free survival, NA = not available, TAH = total abdominal hysterectomy, UTROSCT = uterine tumor resembling an ovarian sex-cord tumor.

### 2.1. Case 1: cotyledonoid-dissecting leiomyoma

**Figure 1.**
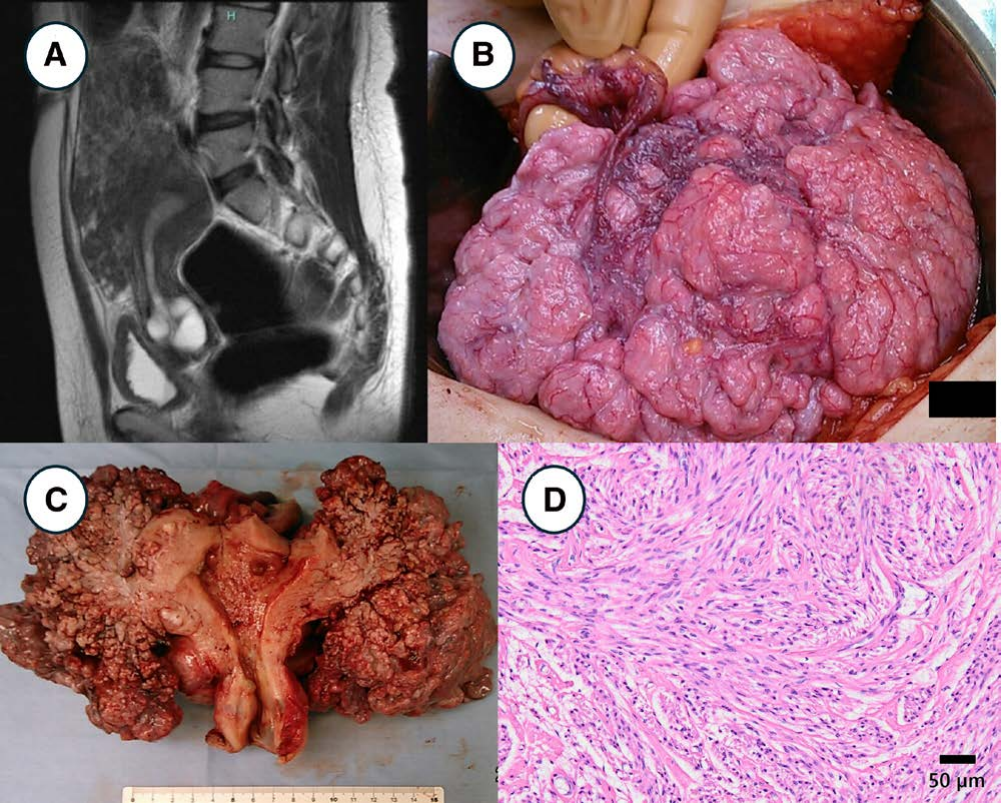
Clinicopathological imaging of case 1. (A) T2-weighted MRI showed the uterine tumor showed heterogeneous signals and an unclear border with the normal uterus. (B) Intraoperative findings and (C) resected specimen showed a placenta-like tumor growing outward from the uterus. (D) Smooth muscle cells, without atypia and necrosis, were proliferating in a solid and bundle-like pattern.

A 42-year-old woman presented with lower abdominal pain. She was admitted to the Oita University Hospital for evaluation of an abdominal mass. Plain pelvic magnetic resonance imaging (MRI) revealed a 17 cm irregular mass with abundant blood flow in the pelvis (Fig. 1A). The border between the tumor and the normal uterine myometrium was unclear, but diffusion-weighted imaging showed no diffusion restriction. Cervical and endometrial cytological tests were negative for malignancy. Total abdominal hysterectomy (TAH) and bilateral salpingo-oophorectomy (BSO) were performed for the preoperative diagnosis of uterine sarcoma. Intraoperative findings showed a 20 cm mass that grew exophytically from the anterior wall of the uterus, with a cauliflower-like or placenta-like shape, and was abundantly vascularized (Fig. 1B and C). The tumor had grown in the retroperitoneal space on the right side of the uterus. Histological examination revealed solid proliferation of smooth muscle cells without atypia or necrosis (Fig. 1D). The final diagnosis established was cotyledonoid-dissecting leiomyoma.

### 2.2. Case 2: uterine tumor resembling ovarian sex-cord tumor (UTROSCT)

**Figure 2.**
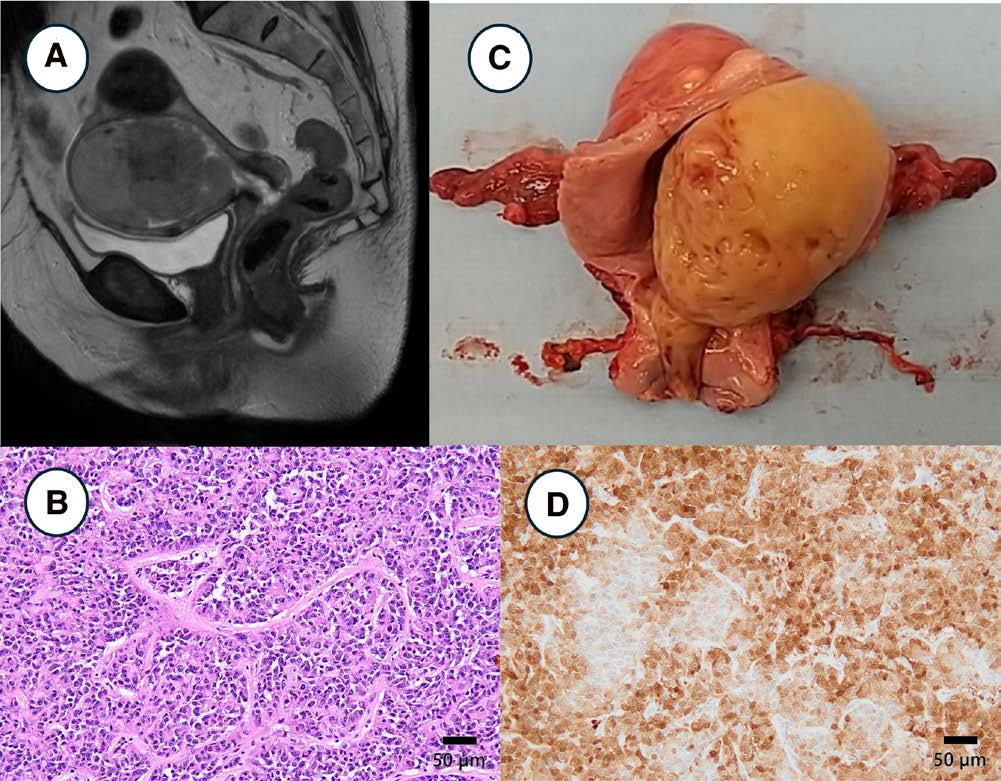
Clinicopathological imaging of case 2. (A) T2-weighted MRI showed a tumor with a heterogeneous signal occupying the uterine cavity. (B) The resected specimen showed a pedunculated mass arising from the posterior uterine wall. (C) Histologically, the tumor cells showed solid, corded, and pseudoglandular patterns. (D) Immunohistochemically, the tumor is positive for calretinin.

A 59-year-old woman presented with abnormal genital bleeding. She was admitted to the Oita University Hospital for evaluation of a uterine mass. Pelvic contrast MRI revealed a 10 cm mass in the uterine cavity with multiple cystic structures. The tumor was suspected to have spread to the cervical and upper vaginal cavities (Fig. 2A). The solid area shows marked diffusion restriction on diffusion-weighted imaging and contrast enhancement on dynamic contrast imaging. Cervical cytology revealed atypical glandular cells, and an endometrial biopsy revealed diffuse proliferation of atypical cells (Fig. 2B). Immunohistochemically, the tumor was positive for the epithelial markers AE1/AE3 and the mesenchymal marker vimentin, suggesting carcinosarcoma. TAH, BSO, retroperitoneal lymphadenectomy, and omentectomy were performed. Macroscopic examination of the resected specimen revealed a tumor originating from the posterior wall of the uterus, occupying the uterine cavity, and descending to the cervix (Fig. 2C). Histological findings showed that cells with mild atypia grew in diffuse, corded, tubular, and pseudoglandular patterns. Immunohistochemically, the tumor showed positive for AE1/AE3, CAM5.2, estrogen receptor, progesterone receptor, desmin, WT1, calretinin (Fig. 2D), vimentin, p16, p53, inhibin (partially), and α-smooth muscle actin (partially) and negative for CD10, cyclinD1, CD117, MyoD1, myogenin, caldesmon, SF1, CD99, SOX10, and HMB45. The patient was diagnosed with UTROSCT.

### 2.3. Case 3: metastasizing leiomyoma

**Figure 3.**
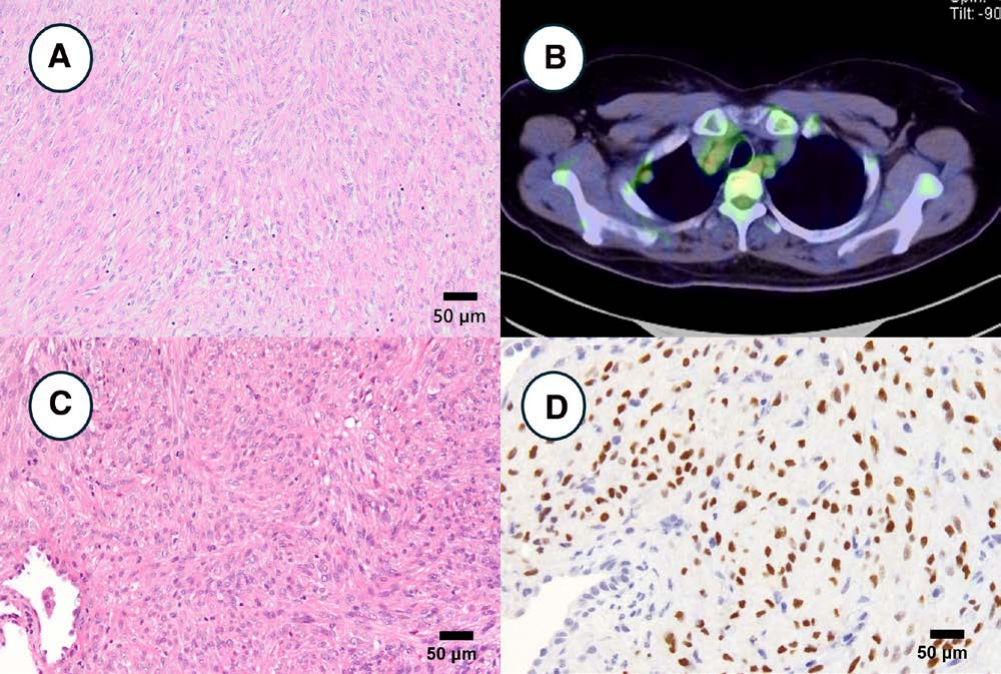
Clinicopathological imaging of case 3. (A) Histologically, the uterine tumor consisted of smooth muscle cells without atypia and necrosis. (B) PET-CT showed that the lung tumor had minimal nonspecific FDG uptake. (C) Histologically, the lung tumor consisted of smooth muscle cells without atypia and necrosis. (D) Immunohistochemically, the lung tumor had diffused positivity for estrogen receptor.

A 53-year-old woman was diagnosed with a pulmonary mass during a routine health examination. At 42 years of age, TAH was performed for hypermenorrhea secondary to a submucosal leiomyoma. The pathological diagnosis was a uterine leiomyoma (Fig. 3A). Eleven years after the operation, a right partial pneumonectomy was performed at the Oita University Hospital for suspected right upper lobe lung cancer detected on computed tomography (CT). Preoperative Positron emission tomography (PET-CT) scan revealed that the lung tumor had a minimal and nonspecific 18-fluoro-deoxy-glucose (FDG) uptake (Fig. 3B). Histological findings revealed smooth muscle cells without atypia and necrosis with a solid pattern (Fig. 3C). Immunohistochemical analyses depicted that the tumor was positive for estrogen receptor (Fig. 3D), progesterone receptor, desmin, and α-smooth muscle actin, but negative for S100. The tumor showed no signs of necrosis. Based on the patient’s history, the pathological diagnosis was leiomyoma, and the diagnosis was benign metastasizing leiomyoma (BML).

### 2.4. Case 4: torsion of a subserosa uterine leiomyoma

**Figure 4.**
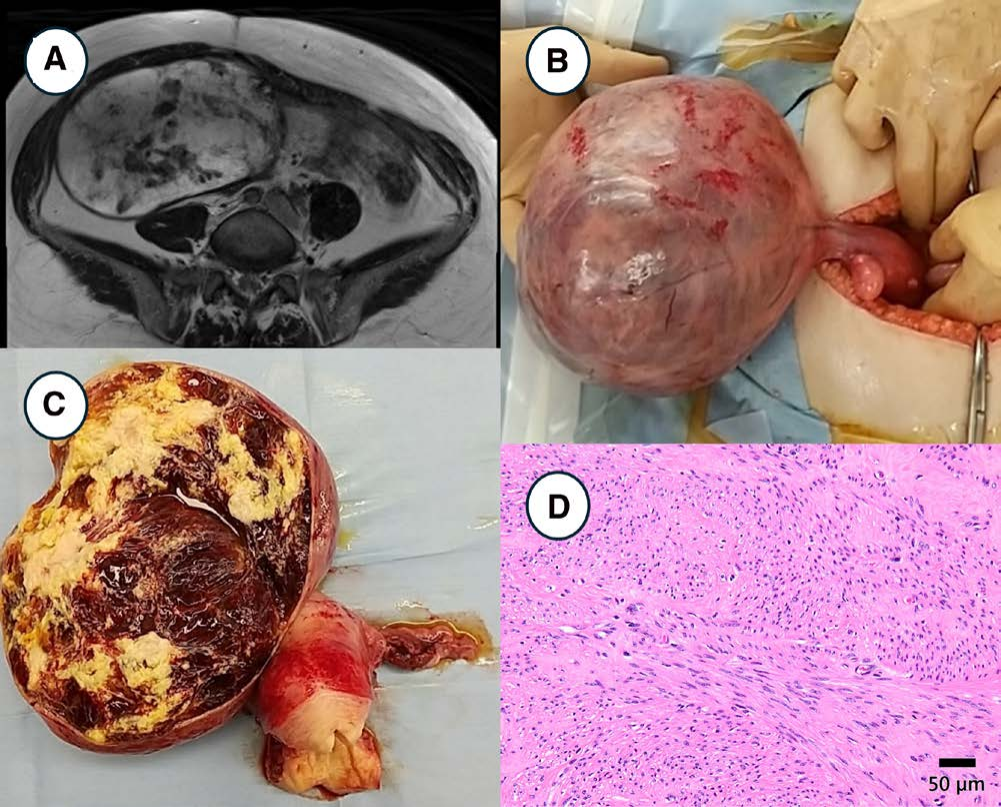
Clinicopathological imaging of case 4. (A) T2-weighted MRI showed a solid mass with a heterogeneous signal in the location of the right adnexa. (B) Intraoperative findings and (C) resected specimen identified a pedunculated mass of uterine origin with intertumoral bleeding and degeneration. (D) Smooth muscle cells, without atypia and necrosis, were proliferating in a solid, bundle-like pattern.

A 50-year-old woman presented with abdominal fullness but no pain. She visited Oita University Hospital for a pelvic tumor examination. Contrast-enhanced pelvic MRI revealed multiple uterine fibroids and a 159 mm right ovarian mass (Fig. 4A). The ovarian mass shows heterogeneous signals on T2-weighted images. The TAH and BSO tests were performed. Intraoperative findings showed that the mass, which was thought to be an ovarian mass, was a pedunculated mass continuous with the uterus, with the stalk twisted 180° (Fig. 4B). The pathological diagnosis was a uterine leiomyoma with degeneration and bleeding (Fig. 4C) and torsion of a subserosal uterine leiomyoma (Fig. 4D).

## 3. Discussion and conclusions

The present study showed that unfamiliarity with uterine mesenchymal tumors may lead to overdiagnosis and overtreatment. At our institution, the diagnostic workflow routinely includes transvaginal ultrasound, gynecologic pelvic examination, systemic CT, pelvic MRI (including diffusion-weighted imaging), cervical cytology/biopsy, and endometrial cytology/biopsy for suspected gynecologic malignancies. The information is evaluated by a gynecologic oncologist, radiologists, pathologists, and cytopathologists to determine the diagnosis. If the diagnosis remains uncertain, multidisciplinary discussions are conducted prior to surgery. However, overdiagnosis and overtreatment can still occur. Physicians must be aware, and also explain to patients, that preoperative clinical imaging and pathological biopsy cannot completely resolve these diagnostic pitfalls.^[8]^ Lok et al reported that an intraoperative pathological diagnosis may be useful for diagnosing uterine mesenchymal tumors.^[9]^ However, this study was limited to uterine smooth muscle tumors and did not consider endometrial stromal tumors or UTROSCT.^[9]^ The risk of over-^[10]^ and underestimation^[7]^ by intraoperative pathological diagnosis has been shown in endometrial stromal tumors. Additionally, Zhao et al revealed that intraoperative pathological diagnosis was not useful for ovarian sex-cord-stromal tumors, which are the ovarian counterparts of UTROSCT.^[11]^ The characteristics of each tumor and important diagnostic points to avoid pitfalls are described below.

Cotyledonoid-dissecting leiomyoma exhibits exophytic growth and irregular nodular dissection of bland smooth muscle cells within the myometrium.^[12,13]^ During the reproductive years, tumor resection with uterus-preserving surgery is feasible because of its favorable clinical outcomes.^[12]^ However, owing to the sarcomatoid appearance of the lesion during surgery, overtreatment is often performed in clinical practice.^[13]^ A placenta-like appearance is the most important feature of this rare tumor.^[12,13]^ If these characteristic intraoperative findings are observed, the uterine preservation can be considered, provided that intraoperative pathological findings confirm a smooth muscle tumor without atypia and necrosis.^[12]^

UTROSCTs are benign in most cases but should be considered to have a low malignant potential because they may recur.^[14–17]^ One of the largest studies about UTROSCTs reports a recurrence rate of approximately 8%.^[15]^ They exhibit various morphological patterns (diffuse, corded/trabecular, tubular, sertoliform, fascicular, whorled, nested, microfollicular, and pseudoglandular), often in combination.^[14,15]^ Therefore, UTROCSTs tend to be pathologically misdiagnosed with other uterine malignancies such as endometrial carcinoma, carcinosarcoma, and uterine sarcoma.^[16,17]^ Notably, finding positivity for sex-cord-stromal markers (inhibin, calretinin, CD99, WT1, FOXL2, and melan A) can suggest this condition.^[14,15,17]^ Even if a UTROSCT is correctly diagnosed, whether the tumor should be treated clinically as benign or malignant remains controversial. Recent studies have also suggested the use of clinicopathological markers. A large tumor size, frequent mitotic figures, necrosis,^[15,17]^ and GREB1::NCOA2 fusion genes^[14,16]^ may be risk factors for recurrence.

Most metastasizing leiomyomas have an indolent course; however, extensive disease may lead to respiratory failure and death.^[3,18]^ Metastasizing leiomyomas are hormonally sensitive and may respond to progestin-, aromatase-, and gonadotropin-releasing hormone agonists/antagonists.^[18]^ Saadeh et al reported that none of the pulmonary nodules showed metabolic uptake of FDG in the total body PET-CT scan, although the primary tumor in the uterus was FDG-avid in 2 patients.^[19]^ Moreover, in 33 of the 36 cases of metastasizing leiomyomas in whom PET scans were previously reported, there was little or no FDG uptake, similar to the present case 3.^[19]^ Notably, in one of the remaining 3 cases that did show uptake, the tumor showed aggressive behavior.^[19,20]^ PET-negative metastasis helps differentiate BML from sarcomas or other malignancies. When a PET scan is negative for metastasis, physicians may consider forgoing a biopsy.

Torsion of a subserosal uterine leiomyoma is a rare cause of acute abdomen.^[21–25]^ This lesion can mimic ovarian cancer by exhibiting necrosis, edema, bleeding, and cystic or ischemic changes. Although the diagnosis of torsion in subserosal uterine leiomyomas is typically confirmed intraoperatively, clinical imaging can provide valuable preoperative information.^[23]^ Doppler ultrasonography can detect a reduced blood supply when the vascular pedicle is visible.^[24]^ Contrast-enhanced CT is useful in excluding other conditions related to acute pain.^[21]^ MRI contributes significantly to the diagnosis of twisted uterine leiomyoma by the pedicle connecting the uterus to the necrotic leiomyoma.^[25]^

In conclusion, we highlighted the pitfalls linked to rare uterine mesenchymal tumors. Detailed pre- and intraoperative clinical and pathological evaluations may not be sufficient for diagnosis. Physicians should be aware of the diversity of uterine mesenchymal tumors and the difficulty in their diagnosis to improve clinical decision-making.

## Acknowledgments

We would like to thank Editage (www.editage.jp) for the English language editing.

## Author contributions

**Conceptualization:** Shizuka Iwabuchi, Mitsutake Yano, Eiji Kobayashi.

**Data curation:** Shizuka Iwabuchi, Yusuke Sato, Mamiko Okamoto, Mitsutake Yano, Eiji Kobayashi.

**Formal analysis:** Shizuka Iwabuchi, Yusuke Sato, Mamiko Okamoto, Mitsutake Yano, Eiji Kobayashi.

**Funding acquisition:** Mitsutake Yano.

**Investigation:** Shizuka Iwabuchi, Yusuke Sato, Mamiko Okamoto, Mitsutake Yano, Eiji Kobayashi.

**Methodology:** Shizuka Iwabuchi, Yusuke Sato, Mamiko Okamoto, Mitsutake Yano, Eiji Kobayashi.

**Project administration:** Shizuka Iwabuchi, Mitsutake Yano, Eiji Kobayashi.

**Resources:** Shizuka Iwabuchi, Yusuke Sato, Mamiko Okamoto, Mitsutake Yano, Eiji Kobayashi.

**Software:** Mitsutake Yano.

**Supervision:** Mamiko Okamoto, Mitsutake Yano, Eiji Kobayashi.

**Validation:** Shizuka Iwabuchi, Yusuke Sato, Mamiko Okamoto, Mitsutake Yano, Eiji Kobayashi.

**Visualization:** Shizuka Iwabuchi, Yusuke Sato, Mamiko Okamoto, Mitsutake Yano.

**Writing – original draft:** Shizuka Iwabuchi, Mitsutake Yano.

**Writing – review & editing:** Shizuka Iwabuchi, Yusuke Sato, Mamiko Okamoto, Mitsutake Yano, Eiji Kobayashi.
